# SOHLHs Might Be Gametogenesis-Specific bHLH Transcriptional Regulation Factors in *Crassostrea gigas*

**DOI:** 10.3389/fphys.2019.00594

**Published:** 2019-05-15

**Authors:** Guang Qian, Yongbo Bao, Danli Song, Na Chen, Zhihua Lin

**Affiliations:** ^1^Zhejiang Key Laboratory of Aquatic Germplasm Resources, College of Biological and Environmental Sciences, Zhejiang Wanli University, Ningbo, China; ^2^School of Marine Sciences, Ningbo University, Ningbo, China

**Keywords:** *Crassostrea gigas*, SOHLH, phylogenetics, gametogenesis, expression

## Abstract

The self-renewal and differentiation of germ cells are essential for gametogenesis and reproduction. In mammals, the transcription factors SOHLH1 and SOHLH2, two members of the bHLH family, are specifically expressed in the gonads, and play an important role in spermatocyte and oocyte differentiation. In our previous study, we performed a phylogenetic analysis of the Lophotrochozoa bHLH genes, and two *Sohlh* were identified in the Pacific oyster *Crassostrea gigas*. Based on the genomes of other species that have complete genomic information, we further analyzed the phylogenetics of the *Sohlh* in this study. The results indicate that the *Sohlh* are ancient genes that were lost in many species during evolution, including in some invertebrates, and lower vertebrates. The phylogenetic tree shows that Sohlh1 and Sohlh2 are located in different scaffolds and that they have low similarity, suggesting early separation in invertebrates. We used RNA-seq and RT-PCR to examine the mRNA expression of the *Sohlh* in *C. gigas* (termed *Cg-Sohlh*), we found that *Cg-Sohlh1*, and *Cg-Sohlh2* are specifically expressed in the gonads. During gonadal development, the mRNA expression levels of both genes increased from the proliferative stage and reached the highest level at the growth stage (*P* < 0.05). Then, the expression level decreased until the resting stage. In addition, immunohistochemistry was used to determine that the Cg-SOHLH1 protein was specifically expressed in the spermatogonia and spermatocytes. *Cg-Sohlh2* mRNA was expressed in both the male and female gonads, while *Cg-Sohlh1* mRNA was highly expressed in the female gonads at all developmental stages except for the resting stage. These data indicate that Cg-SOHLH might be gonad-specific regulatory factors, similar to mammalian SOHLH, and that Cg-SOHLH1 might be involved in spermatogonial differentiation. This study lays the foundation to further determine the functional role of SOHLH in mollusk gametogenesis and provides a foundation to better understand the regulatory mechanism of gametogenesis in invertebrates.

## Introduction

Basic helix-loop-helix (bHLH) proteins constitute a large transcriptional regulator family that is characterized by the possession of a bHLH domain involving DNA binding, which plays critical roles in the regulation of biological growth and development (Wang et al., 2010). For example, some transcription factors, such as MyoD, Olig and Neurogenin, act as key regulators of islet cells during embryonic development (Schwitzgebel et al., 2000), while others, such as Bmal, Clock, and HIF, play critical roles in the circadian clock (Bmal, Clock) or the response to hypoxia (HIF) (Ivan et al., 2001; Rudic et al., 2004). The transcription factors SOHLH1 and SOHLH2, two members of the bHLH family, are expressed in the testes and early ovaries and are required for spermatogonia differentiation, oocyte differentiation and primordial follicle development in mammals (Toyoda et al., 2014). SOHLH1 and SOHLH2 were expressed in adult testes of A spermatogonia throughout the differentiation stages (Ballow D. et al., 2006; Ballow D.J. et al., 2006), whereas in mouse oogenesis, the *Sohlh1* and *Sohlh2* transcripts were upregulated shortly after birth, and their encoded proteins were expressed in primordial oocytes in the ovaries (Pangas et al., 2006; Shin et al., 2017). Moreover, *Sohlh1*-knockout and *Sohlh2*-knockout mice had common phenotypes, exhibiting a massive accumulation of spermatogonia, a reduction of mature spermatozoa in the testes (Hao et al., 2008), and an interruption of primary follicle development in the ovaries (Choi et al., 2008).

The Pacific oyster, *Crassostrea gigas*, is a successive, and irregular protandrous hermaphrodite mollusk (Guo et al., 1998). In the adult, the gonad is a diffuse organ made of numerous tubules that are separated by connective tissue, and the whole organ constitutes the gonadic area (Franco et al., 2008); the annual reproductive cycle is subdivided into five stages as follows: the proliferation stage, growing stage, maturation stage, emission stage, and resting stage (Heudeberthelin et al., 2001). The gonadal structure of *C. gigas* is formed by gonadal tubules invaginated in connective tissue that constitutes the storage tissue of male and female gonads. Spermatogonia, spermatocytes and spermatids are observed in the male gonads, according their nucleus diameter, and aspect ratio (Franco et al., 2008). In the female gonads, oocyte development is a major event in gametogenesis, and the types of oocytes are identified based on those diameters (Lango-Reynoso et al., 2000).

In our previous study of bHLH gene taxonomy and phylogenetic analysis in Lophotrochozoa, two *Sohlh* were found in mollusks, but they were lost in other lower invertebrates or were only found in one type of species, such as birds, reptiles, and amphibians. It would be interesting to study their evolution and function (Bao et al., 2017). In this study, we analyzed the phylogenetics of SOHLH1 and SOHLH2 based on the genomic information of species that have completed genome sequencing information. To investigate the functional role of SOHLH in *C. gigas*, the mRNA expression of *Cg-Sohlh* was examined in different adult tissues, and their expression patterns in the gonads at different developmental stages were also analyzed. Moreover, the expression of the SOHLH1 protein was further analyzed in the male gonads at three stages (growing stage, maturation stage, and resting stage). This study lays the foundation to further illustrate the functional role of SOHLH in the regulation of gametogenesis in mollusks and provides the foundation to better understand the regulatory mechanism of gametogenesis in invertebrates.

## Materials and Methods

### Data Set Collection of the Sequences of SOHLH Proteins

The annotated sequences of SOHLH were obtained from NCBI^1^, and the sequences of SOHLH proteins were searched against the SMART^2^ database to identify the bHLH domain. This search was also supplemented using TBLASTN with the sequence of the bHLH domain and was based on the species that have complete genome sequencing information (Supplementary Table S1); searches were stringently performed with *e*-values less than 1e^-3^. All of the collected SOHLH sequences were used for multiple alignments.

### Phylogenetic Analysis

A multiple alignment was performed using MAFFT 7.221 (Katoh and Standley, 2013) with the E-INS-I algorithm for the amino acid sequences of the bHLH domain. The resulting alignment was used to perform maximum likelihood (ML) phylogenetic analyses with the program RAxML (Stamatakis, 2006) using the evolutionary model LG+Gamma+Invariant, and 1,000 replicates were performed to obtain bootstrap support (BS) values. Bayesian inference (BI) phylogenetic analysis was conducted with MrBayes 3.2.2 (Ronquist and Huelsenbeck, 2003). Four Markov chains were run for 3 × 10^6^ generations, and sampling was performed every 100 generations to yield *a posterior* probability distribution of 104 trees. The first 25% of the trees were discarded when compiling the summary statistics and consensus trees.

### Animal Material and Tissue Sampling

Pacific oysters (*C. gigas*) were collected from a marine product farm in Qingdao, Shandong Province, when they were approximately 2 years old. Thirty oysters averaging approximately 12 cm in shell length were sampled every 15 days between May and September 2017 for the recognition of sex and gonadal development by using histological observation. Additionally, five male and five female oysters were selected in each gonadal development stage (proliferative stage, growing stage, maturation stage, emissions stage, and resting stage), and five tissues from those selected oysters, including the hepatopancreas, gill, adductor muscle, mantle and gonads, were collected in liquid nitrogen and then stored at -80°C until further analysis.

### RNA Extraction and cDNA Synthesis

The total RNA was extracted using TRIzol Reagent (TaKaRa, Japan) according to the manufacturer’s instructions. The RNA concentration was measured on a Nanodrop Spectrophotometer (Thermo Fisher Scientific). RNA degradation and contamination were monitored with 1% agarose gels. These RNA samples conformed to the required purity criteria (A260/A230 and A260/A280 > 1.80) for cDNA synthesis using the PrimeScript^TM^ RT Reagent Kit with gDNA Eraser (Perfect Real Time) (TaKaRa, Japan) according to the manufacturer’s instructions.

### Real-Time Quantitative PCR

The *Cg-Sohlh* mRNA tissue expression and gonadal development were analyzed by RT-PCR and qRT-PCR, respectively. The PCR primer information is shown in Table 1. SYBR Green detection chemistry (TaKaRa, Japan) was performed in an ABI 7500 Fast PCR System (Thermo Fisher Scientific, United States) for qRT-PCR experiments. The internal control was 18S rRNA to normalize the *Sohlh* mRNA for quantification. PCR was carried out in a total volume of 20 μl and contained 2 μl of cDNA, 2 μl of each primer (10 mM), 6 μl of RNase-free water, and 10 μl of the SYBR Green PCR Master Mix (TaKaRa, Japan). The cycling conditions were 94°C for 5 min, followed by 40 cycles of 94°C for 15 s, and 60°C for 40 s. At the end of the PCR cycles, melting curve analyses were performed. The Ct value was defined as the fractional cycle number at which the fluorescence passes the fixed threshold. Each sample was analyzed in triplicate by PCR.

**Table 1:** Primer sequence information in the current study.

Primer	Sequence (5′-3′)	Used for
Cg-Sohlh1 F1	CTGAAACTGGTCAACGAAGC	RT-PCR
Cg-Sohlh1 R1	TGGTGGAGGTGTAAAAGGG	
Cg-Sohlh1 F2	CCTTTTACACCTCCACCACC	qRT-PCR
Cg-Sohlh1 R2	CATAATCTGAGACCCCTGCTATG	
Cg-Sohlh1 F3	GGATCCGGAGAGGTTCTGTTGGTTGT	ORF amplification
Cg-Sohlh1 R3	CTCGAGATGTCACTAGGTTGGATTCTC	
Cg-Sohlh2 F1	CAGCGAGAATGGGGTGTAT	RT-PCR
Cg-Sohlh2 R1	CTTGTCGAGGTCTTGGGAGT	
Cg-Sohlh2 F2	GAGAGCAACCCAATCAGAAAC	qRT-PCR
Cg-Sohlh2 R2	AGCAGTCCTCACATAGAGCAAC	
Cg-Sohlh2 F3	GAATTCATGTCTTTAGCGGTACAAGAGATC	ORF amplification
Cg-Sohlh2 R3	CTCGAGATACGGACTATACTTCAGAAGG	
Cg-18sRNA F	TTGGTTTTCGGAACACGAGGTAAT	qRT-PCR
Cg-18sRNA R	TGCTTGGCAAATGCTTTCGCTG	


### Antibody Preparation

A Cg-SOHLH1 polyclonal antibody was prepared against our recombinant protein. The sequence of ORF was amplified using enzyme site-specific primers (Table 1) and was cloned into the pET-28a vector. The recombinant vector was transformed into DH5α cells (TaKaRa, Japan) for amplification and then transformed into BL21 (DE3) cells (TaKaRa, Japan) for protein expression. The expressed protein was purified by a Ni-NTA column using Ni-NTA Sefinose^TM^ Resin (BBI, United States) and was sent to Sangon Bio Company for antibody preparation. The purified protein was injected into rabbits for polyclonal antibody production, and the serum of rabbits was tested by ELISA each week during immunization. After 6 weeks of immunization, the antiserum was harvested from the rabbits, and the antibody was purified and stored at -20°C after western blot testing.

### Immunohistochemical Detection

For the immunohistochemical assay, the gonads of male oysters in 3 stages (growing stage, maturation stage, and resting stage) were fixed in Bonn’s solution. After dehydration with xylene, they were embedded in paraffin for sectioning. Two 5-mm-thick consecutive sections were cut from each sample and were mounted on poly-lysine-coated slides. One section was used for immunohistochemical staining of Cg-SOHLH1 and the other was used for PAS–hematoxylin staining. After deparaffinizing and rehydrating the sections with xylene and a series of ethanol dilutions, the sections were washed twice with TBS buffer (10 mM Tris–HCl, pH 8.0, 100 mM NaCl) for 5 min each, followed by antigen retrieval by microwaving the slides at 700 W for 15 min in a 10 mM sodium citrate solution, pH 6.0. After three washes with ddH_2_O and TBS, the sections were incubated at room temperature for 1 h in a blocking solution (TBS containing 1% BSA and 3% goat serum). After blocking, the sections were incubated with a Cg-SOHLH1 antibody diluted at 1:200 in 5% bovine serum albumin (BSA) at 4°C overnight. Then, the sections were washed three times with TBS and were incubated with an HRP-labeled anti-rabbit IgG antibody (1:3000) in 5% BSA for 45 min at room temperature. After washing three times with TBS, 100 μl of DAB, a color reagent, was applied and developed at room temperature for 3–30 min. The development time was controlled under a microscope and the sections were rinsed with distilled water.

### Statistical Analysis

The mRNA expression levels of the *Cg-Sohlh* were calculated with the 2^-ΔΔCT^ method, and the n-fold change relative to the corresponding control represented the expression value. The statistical significance differences between the experimental and control groups were determined using one-way analysis of variance (ANOVA), which was followed by multiple Duncan tests, and the data are shown as the mean ± SD. Any significant differences are indicated with an asterisk at *P* < 0.05 and two asterisks at *P* < 0.01.

## Results

### Structural Characteristics of *Cg-Sohlh* Genes

**FIGURE 1:**
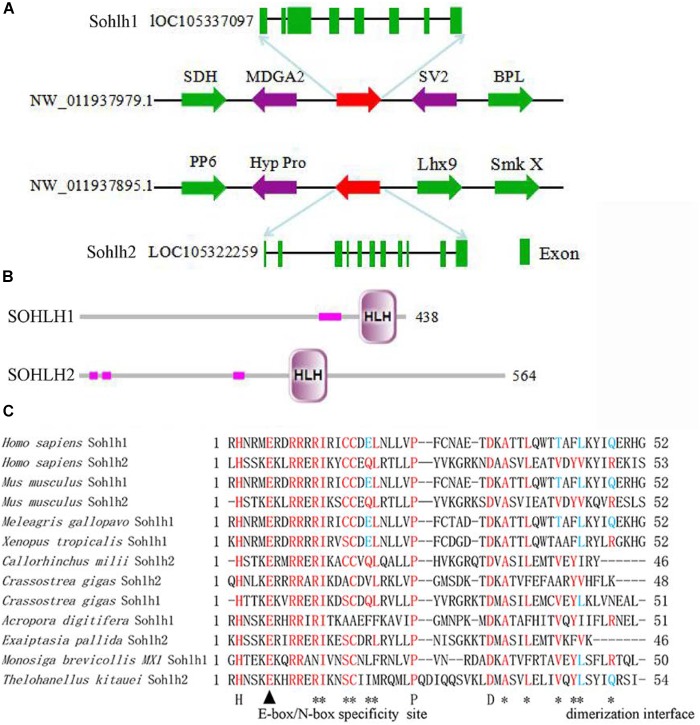
Structural analysis of the *Cg-Sohlh* genes. **(A)** The location and exon information of the *Cg-Sohlh* genes are shown. **(B)** The structures of the *Cg-Sohlh* genes are shown; the small pink rectangles represent low complexity regions. **(C)** The bHLH domain sequence alignment of *Sohlh* genes in different species is shown; bHLH specificity site: E-box/N-box (▲), dimerization interface (_∗_).

The SOHLH sequences of *C. gigas* were acquired according to our previous study (Bao et al., 2017), namely, Cg-SOHLH1 (XP_011439966), and Cg-SOHLH2 (EKC27190.1). The structural analysis of the *Cg-Sohlh* genes showed that there were 8 exons in *Sohlh1* and 11 exons in *Sohlh2* and that they were located on different scaffolds in the genome (Figure 1A). A SMART analysis showed that both the *Cg-Sohlh1* and *Cg-Sohlh2* genes had a basic helix1-loop-helix2 (bHLH) domain (Figure 1B) and that they were conserved in the bHLH domain with the E-box/N-box specificity site and in the dimerization interface sites among species (Figure 1C). These sites can be combined with DNA in a helix1-loop-helix2 of bHLH proteins.

### The Distribution of *Sohlh* Genes in Species

A total of 111 annotated genes among 93 species were obtained by using protein blast searches in NCBI, including 52 SOHLH1, 40 SOHLH2, and 19 uncharacterized genes (Supplementary Table S2). The unannotated SOHLH sequences were compared to sequenced genomic species by using tblastn and were then arranged to analyze their distributions, as shown in Table 2. SOHLH originate from Cnidaria, but they are absent in many species, including most of the lower invertebrates (Nematoda, Rotifera, Tardigrada, Nematomorpha, and Platyhelminthes), Echinodermata, and lower vertebrates (Leptocardii and Ascidiacea).

**Table 2:** The distribution of SOHLH in genome-sequenced species.

Phylum	Class	SOHLH1	SOHLH2	Phylum	Class	SOHLH1	SOHLH2
Protozoa	Aconoidasida	–	–	Arthropoda	Chilopoda	–	–
	Sarcodina	–	–		Remipedia	–	–
	Flagellata	–	–		Merostomata	+	–
Placozoa	Trichoplax	–	–		Branchiopoda	–	–
Porifera	Demospongiae	–	–		Arachnida	+	–
Cnidaria	Anthozoa	+	+		Insecta	–	–
	Hydrozoa	+	+	Tardigrada	Eutardigrada	–	–
	Myxosporea	–	–	Echinodermata	Holothuroidea	–	–
Ctenophora	Tentaculata	–	–		Echinoidea	–	–
Platyhelminthes	Turbellaria	–	–	Brachiopoda	Lingulata	+	–
	Cestoda	–	–	Hemichordata	Enteropneusta	+	–
	Trematoda	–	–	Chordata	Hyperoartia	+	+
Rotifera	Bdelloidea	–	–		Ascidiacea	–	–
Nematoda	Secernentea	–	–		Leptocardii	–	–
	Chromadorea	–	–		Chondrichthyes	+	+
	Enoplea	–	–		Actinopterygii	+	–
Mollusca	Bivalvia	+	+		Amphibia	+	–
	Gastropoda	+	+		Reptilia	+	–
	Cephalopoda	+	+		Aves	+	–
Annelida	Clitellata	–	–		Mammalia	+	+
	Polychaeta	–	+				


*+, existence; -, no existence.*

### Phylogenetic Analysis

**FIGURE 2:**
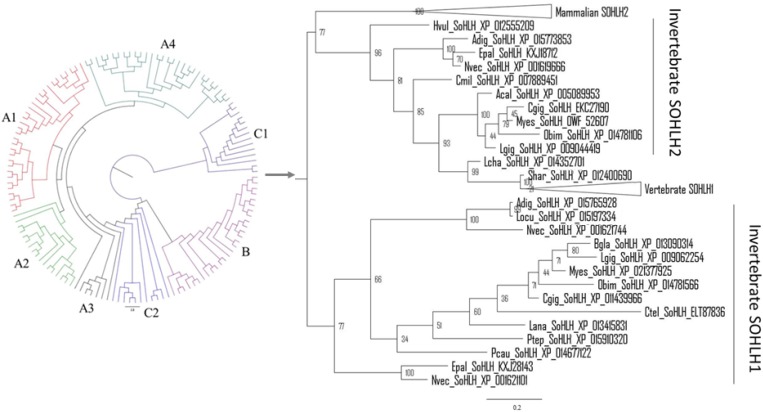
Molecular phylogenetic analysis of SOHLH sequences. The phylogenetic analysis was constructed based on the bHLH domain of SOHLH by MrBayes. A1, mammalian SOHLH1; A2, aves SOHLH1; A3, reptilian SOHLH1; A4, fish SOHLH1; B, mammalian SOHLH2; C1, invertebrate SOHLH1; C2, invertebrate SOHLH2. The same tree with branch lengths, support values and gene names, is shown in Supplementary Figures S3, S4.

The phylogenetic tree was constructed based on the bHLH domain of the collected SOHLH sequences. As shown in Figure 2, the invertebrate SOHLH2 gene clustered as a large branch with the vertebrate SOHLH1 and SOHLH2 genes, and Cg-SOHLH1 clustered together with invertebrate SOHLH1 independently of the other SOHLH. Further, we also constructed the evolutionary tree using the full sequence of the representative *Sohlh*. The results of SOHLH1 and SOHLH2 were consistent; in other words, the SOHLH of vertebrates and invertebrates were located in their respective clade in the phylogenetic tree (Supplementary Figures S1, S2). Cg-SOHLH1 showed maximal identity (23%) with vertebrate SOHLH1. By comparison, the invertebrates SOHLH2 genes were more similar to those of vertebrates, and the identity of Cg-SOHLH2 to vertebrate SOHLH2 ranged between 19 and 35%.

### RNA-Seq Analysis of *Cg-Sohlh*

**FIGURE 3:**
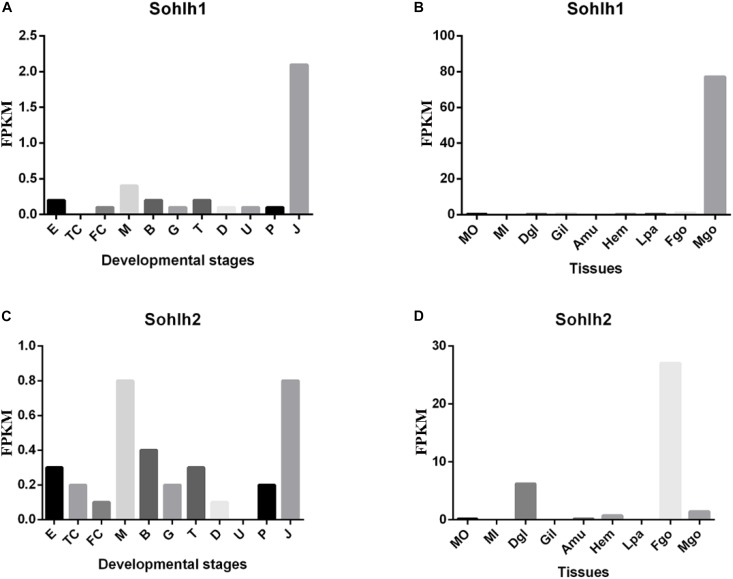
Transcriptional expression of *Cg-sohlh* in developmental stages **(A,C)** and adult tissues **(B,D)**. **(A,C)** The indicated developmental stages are described as follow. E, egg; TC, two cells; FC, four cells; M, morula; B, blastula; G, gastrula; T, trochophore; D, D-larva; U, umbo larva; P, pediveliger; and J, juvenile; B and D, organs are abbreviated as follow. Mo, the outer edge of mantle; Mi, the inner pallial of mantle; Dgl, digestive gland; Gil, gills; Amu, adductor muscle; Hem, hemocyte; Lpa, labial palp; Fgo, female gonad; Mgo, male gonad.

The transcriptome data from multiple adult organs and developmental stages of *C. gigas* were obtained from the NCBI gene expression omnibus (accession GSE31012) and from the supplementary materials of the associated publication (Zhang et al., 2012). The results are shown in Figure 3. In *C. gigas* of the different developmental stages, Cg-*Sohlh1* was highly expressed in juveniles but showed lower expression in other stages, while Cg-*Sohlh2* was highly expressed in the morula stage and in juveniles. In adult *C. gigas*, *Cg-Sohlh1* was highly expressed in the male gonads but was hardly expressed in other tissues, while *Cg-Sohlh2* was highly expressed in the female gonads and showed a certain amount of mRNA expression in the digestive glands and male gonads.

### RT-PCR Analysis of *Cg-Sohlh*

**FIGURE 4:**
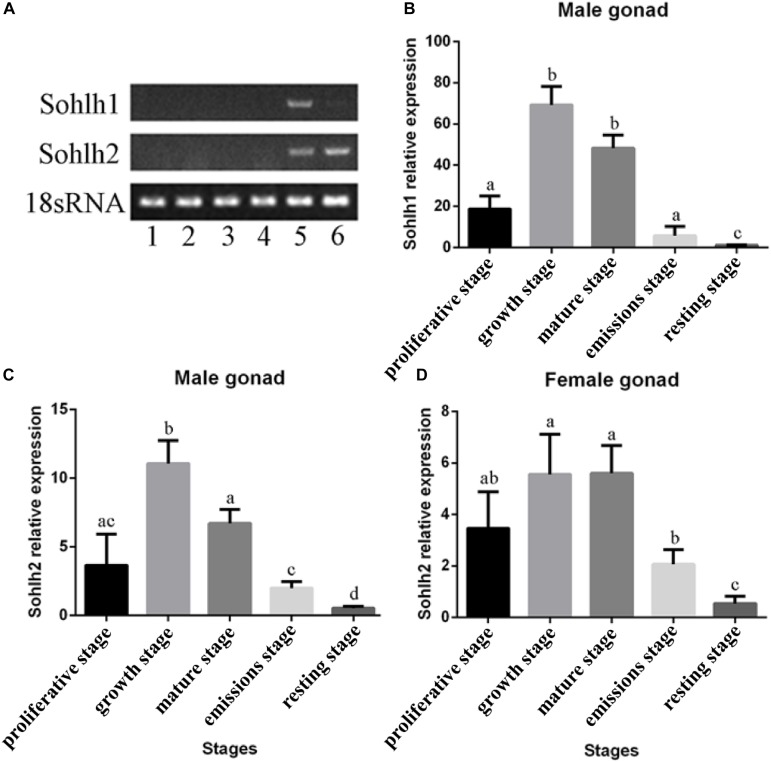
Expression analysis of the transcriptional regulator SOHLH in *Crassostrea gigas*. **(A)** The mRNA expression of *Sohlh* in different adult tissues was determined by RT-PCR. 1, digestive gland; 2, gill; 3, adductor muscle; 4, mantle; 5, male gonad; 6, female gonad. **(B–D)** The *Sohlh* mRNA expression is shown during gonadal development. The expression levels were calibrated against the resting stage (control). The vertical bars represent the mean ± SD., *n* = 5. “a, b, and c” indicate significance to each other (*p* < 0.05).

The mRNA expression patterns of *Cg-Sohlh* are shown in Figure 4. A tissue distribution analysis showed that the *Cg-Sohlh1* and *Cg-Sohlh2* mRNA transcripts were detected in the male gonads, and *Cg-Sohlh2* was also detected in the female gonads; but, neither gene was observed in other tissues, including the digestive glands, gills, adductor muscle, and mantle. Moreover, during five developmental stages, the mRNA expression levels of *Cg-Sohlh1* and *Cg-Sohlh2* increased from the proliferative stage, reached the highest level at the growth stage (*P* < 0.05), and decreased until the resting stage.

### Recombinant Cg-SOHLH Proteins and Antibodies

**FIGURE 5:**
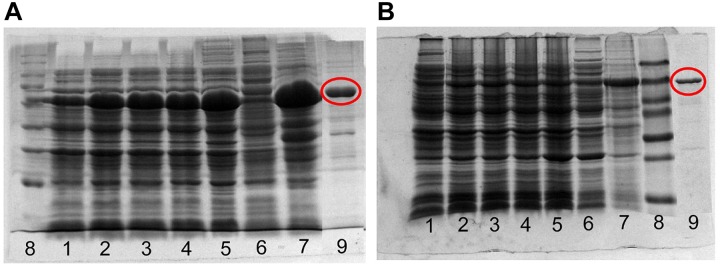
Expression and purification of the recombinant SOHLH protein in oysters. **(A)** SOHLH1 and **(B)** SOHLH2. 1, uninduced; 2–5, induced 3, 4, 5, and 12 h, respectively; 6, supernatant; 7, inclusion body; 8, mark; 9, purification protein. The products of the purified protein are circled in red.

The recombinant expression and purification of the Cg-SOHLH proteins are shown in Figure 5. Both the Cg-SOHLH1 and Cg-SOHLH2 proteins were induced in the inclusion body, and the molecular weights were approximately 70 and 50 kDa, respectively, which was consistent with the previous prediction. Purified proteins were injected into rabbits for polyclonal antibody production. Fortunately, the SOHLH1 protein antibody was successfully generated, but the production of the SOHLH2 antibody failed according to the ELISA and western blot results (Supplementary Figure S5 and Supplementary Table S3).

### Immunohistochemistry Analysis of the Cg-SOHLH1 Protein in the Male Gonads

**FIGURE 6:**
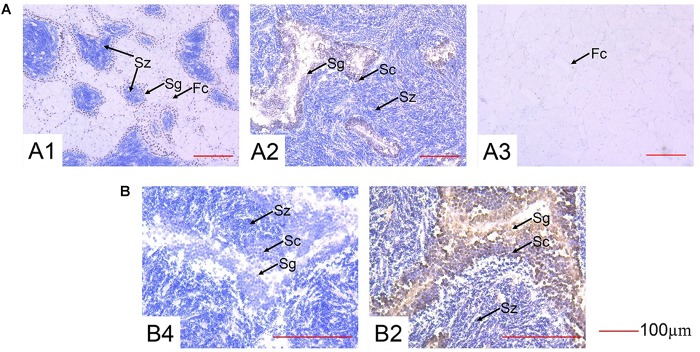
Immunohistochemistry of SOHLH1 protein in the male gonads of oysters. **(A)** 200× magnification. **(B)** 400× magnification. 1, growth stage; 2, maturation stage; 3, resting stage; 4, maturation stage control; Fc, follicular cell; Sg, spermatogonia; Sc, spermatocyte; Sz, spermatozoa.

The localization of the Cg-SOHLH1 protein was detected in the male gonads during three developmental stages, as shown in Figure 6. Four cell types were observed in the male gonads: follicular cells, spermatogonia, spermatocytes, and spermatozoa. The Cg-SOHLH1 protein was specifically detected in spermatogonia and spermatocytes on the side of the follicular wall; however, this protein was not detected in spermatozoa or in follicular cells.

## Discussion

Gametes are an important part of animal growth and development and play a critical role in the reproduction of a species. A large number of studies have reported the process and regulatory mechanism of gametogenesis in higher taxonomic animals, such as mammals (Wolgemuth et al., 1994). SOHLH1 and SOHLH2, two testis- and ovary-specific bHLH transcription factor genes, have recently been reported to be essential for both spermatogenesis (Ballow D. et al., 2006) and oogenesis (Pangas et al., 2006) and are required for spermatogonia differentiation, oocyte differentiation, and primordial follicle development in mammals (Choi et al., 2008; Shin et al., 2017). However, few studies have been conducted on mollusks and other invertebrates. Two SOHLH in mollusks, including *C. gigas*, *Pinctada fucata*, *Lottia gigantea*, *Patella vulgate*, and *Biomphalaria glabrata*, were identified for the first time in our previous study (Bao et al., 2017).

The results of the *Cg-Sohlh* gene structure analysis demonstrated that *Cg-Sohlh1* and *Cg-Sohlh2* are located on different scaffolds in the genome and share only 20% amino acid sequence identity, indicating that there is a low homology between Cg-SOHLH1 and Cg-SOHLH2. However, both had bHLH domains, and their E-box/N-box feature sites and dimer sites are highly conserved. The bHLH motif contains approximately 60 amino acids, consisting of a basic region that can be combined with DNA and a helix 1-loop-helix 2, in which the length of the loop is different in bHLH proteins (Ma et al., 1994). The basic region residues determine the DNA-binding activities of the bHLH proteins (Davis et al., 1990). The binding sites of bHLH define the conserved E-box sequence motif, -CAXXTG-, in which the central 2 bp are specified by each protein (Blackwell and Weintraub, 1990; Alex et al., 1992; Dang et al., 1992). The DNA-binding specificity at the center of the E-box is markedly influenced by a single basic region residue, which is arginine in bHLH zipper proteins (Halazonetis and Kandil, 1991; Antwerp et al., 1992). All of these observations show that the *Cg-Sohlh* genes are members of the bHLH protein family, although they show low homology in the full-length amino acid sequence.

Phylogenetic analysis shows that *Sohlh1* and *Sohlh2* both originate from Cnidaria in invertebrates but are lost in many invertebrates or only one *Sohlh* type is present. Mollusca have both sohlh1 and sohlh2 in the three classes Bivalvia, Gastropoda and Cephalopoda. *Sohlh1* exits in most Chordata, except for the families Ascidiacea and Leptocardii; *Sohlh2* was lost in more classes, including Ascidiacea, Leptocardii, Amphibia, Reptilia, and Aves. To date, few studies have reported why *Sohlh* were lost in some species or which genes replace them to exert the same functions. Cg-SOHLH1 shared no more than 23% identity with the vertebrate SOHLH1 gene, and an evolutionary tree demonstrated that the invertebrate SOHLH1 gene was located in independent branches, indicating that SOHLH1 underwent great genetic variation and a fast evolution rate or that the vertebrate SOHLH1 gene did not evolve from the invertebrate SOHLH1 gene. Cg-SOHLH2 had a higher consistency than that of Cg-SOHLH1 (the identity range was between 19 and 35% for vertebrate SOHLH2) in the phylogenetic tree. The invertebrate SOHLH2 gene formed a large clade with the vertebrate SOHLH1 and SOHLH2 genes, and all SOHLH2 genes followed the species evolutionary relationship, indicating that the invertebrate SOHLH1 and SOHLH2 genes might originate from different ancestral genes or that they separated early in evolution. SOHLH2 was also missing in many classes, such as Aves, Reptilia, Amphibia, Actinopterygii, Leptocardii in vertebrates; and Echinodermata, Tardigrada, Arthropoda, Nematoda, and Platyhelminthes in invertebrates. The multiple loss of SOHLH genes indicates that the function of those genes may be replaced by other genes in some species during evolution.

A tissue mRNA expression analysis showed that *Cg-Sohlh1* was only expressed in the male gonads, and *Cg-Sohlh2* was expressed both in the male and female gonads. In mice, *Sohlh1* and *Sohlh2* were specifically expressed in both testes (undifferentiated and differentiating spermatogonia) (Suzuki et al., 2012) and in the ovaries (early oocyte and primordial follicle) (Shin et al., 2017). Ballow D. et al. (2006) generated *Sohlh1*-KO mice and found that they accumulated PLZF+ spermatogonia and contained few spermatocytes, suggesting they had a defect in spermatogonial differentiation, and the *Sohlh2*-KO mice exhibited the same phenotype as the *Sohlh1*-KO mice (Toyoda et al., 2009). In the ovaries, NOBOX oogenesis homeobox (NOBOX) (Rajkovic et al., 2004) and LIM homeobox protein 8 (LHX8) (Qin et al., 2008), two important regulators of postnatal oogenesis, were coexpressed with SOHLH1. A single deficiency of *Sohlh1* or *Sohlh2* disrupted the expression of LHX8 and NOBOX in the embryonic gonads without affecting meiosis, indicating that *Sohlh1*, and *Sohlh2* are essential regulators of oocyte differentiation but do not affect meiosis I (Shin et al., 2017). Here, together with a phylogenetic analysis, the mRNA expression pattern of *Cg-Sohlh* suggests that *Cg-Sohlh2* might be a homologous gene and had a similar function to the mice *Sohlh*, while *Cg-Sohlh1* was another male-specific gene involved in spermatogenesis.

The temporal expression analysis of *Cg-Sohlh* in five gonadal developmental stages further showed that the expression of *Cg-Sohlh1* in the male gonads increased first and then decreased, with the highest expression at the growth and maturation stages (*p* < 0.05). Similar to mammals, the male *C. gigas* gonadal development undergoes the processes of spermatogonia proliferation and differentiation, which mainly occurs at the growth stage (Franco et al., 2008). Furthermore, we found that the Cg-SOHLH1 protein was expressed in spermatogonia and spermatocytes. All of the above data speculated that the transcription factor SOHLH1 might participate in the differentiation process of spermatogonia in *C. gigas*. The expression trend of *Cg-Sohlh2* in the male gonads was similar to that of *Cg-Sohlh1*. Therefore, we speculated that the transcription factor SOHLH2 might also serve as a regulator in spermatogenesis and oogenesis in *C. gigas*. However, further studies are needed to confirm its functional role.

**FIGURE 7:**
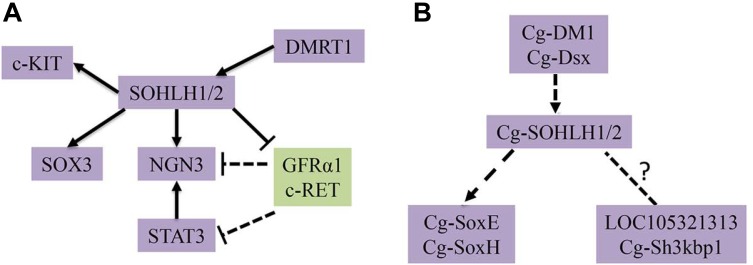
Transcriptional regulatory networks of spermatogonial differentiation. **(A)** The known regulatory network in mammals are shown. **(B)** The predicted regulatory network in *C. gigas* is shown.

SOHLH1 and SOHLH2, two transcription factors, have been reported to participate in spermatogonia differentiation by regulating different target genes in mammals. On one hand, SOHLH1 and SOHLH2 negatively regulate many genes that promote SSC self-renewal, including *c-Ret*, *Gfrα1*, *Nanos2*, and *Pou5f1*, based on a ChIP analysis (Suzuki et al., 2012). On the other hand, those two transcription factors positively regulate other genes that promote SSC differentiation, such as *Ngn3* (Ballow D. et al., 2006) and *Sox3* (Toyoda et al., 2009), according to the binding of the *Ngn3*, and *Sox3* promoter regions (Suzuki et al., 2012). In addition, SOHLH proteins promote later steps of spermatogonial differentiation; this indicates that they both positively regulate c-Kit, which is known to be specifically involved in A-spermatogonia differentiation (Koshimizu et al., 1991; de Rooij et al., 1999). SOHLH1 is also a positive, direct target of DMRT1, which promotes spermatogonial differentiation (Murphy et al., 2010; Matson et al., 2010). We compiled these results and drew a local regulatory network map of spermatogonia differentiation that can be seen in Figure 7. In *C. gigas*, some potential downstream genes of sex determination and differentiation have been identified, such as *Cg-SoxE*, *Cg-Dsx*, and *Cg-SoxH* (Zhang et al., 2012; Santerre et al., 2014), and *Cg-DM1* may be involved in the development of the gonad due to its higher expression in both sexes (Naimi et al., 2009). In addition, Yue et al. (2018) also found some gonad-specific genes by RNA-seq, such as lncRNA LOC105321313 and Cg-Sh3kbp1. Together with our studies on the SOHLH expression pattern, we speculated that there is a potential regulatory network in *C. gigas* that is similar to that in vertebrates (Song and Wilkinson, 2014; Figure 7). All of these identified genes involved in the gametogenesis regulation pathway should be functionally verified in further studies in mollusks.

In conclusion, *Sohlh1* and *Sohlh2* are unstable genes that have been lost multiple times during species evolution, and in some species, their function may be replaced by other genes, including f*c-kit* and *ngn3*. SOHLH1 and SOHLH2 display a low homology in *C. gigas* but show similar expression patterns and functions in gametogenesis. Cg-SOHLH1 is expressed in spermatogonia and spermatocytes and may play a regulatory role in the differentiation of spermatogonia. Cg-SOHLH2 is expressed in both the male and female gonads and, thus, might regulate spermatogenesis and oogenesis. However, the precise physiological function of both factors remains unclear, and this might be illuminated by RNAi and ChIP approaches.

## Author Contributions

YB and ZL conceived and designed the experiments. GQ, DS, and NC performed the experiments and contributed to reagents, materials, and analysis tools. GQ analyzed the data. YB and GQ wrote the manuscript. All authors reviewed the manuscript.

## Conflict of Interest Statement

The authors declare that the research was conducted in the absence of any commercial or financial relationships that could be construed as a potential conflict of interest.

## Footnotes

1 https://www.ncbi.nlm.nih.gov/

2 http://smart.embl-heidelberg.de

## Supplementary Material

FIGURE S1 Phylogenetic analysis of SOHLH1 full sequence in species.

FIGURE S2 Phylogenetic analysis of SOHLH2 full sequence in species.

FIGURE S3 Phylogenetic analysis of bHLH domain of SOHLH1 and SOHLH2 in species.

FIGURE S4 Phylogenetic analysis of bHLH domain of SOHLH1 and SOHLH2 in species with human group A gene outgroups according published article (Bao et al., 2017).

FIGURE S5 Western blot results of CgSOHLH1 and CgSOHLH1antibody detecting. **(A)** The target band of CgSOHLH1 is about 70 kDa. The bands from left to right are marker, recombinant protein and male gonad tissue of oyster. **(B)** The target band of Cg SOHLH2 is about 50 kDa. The bands from left to right are marker, recombinant protein of SOHLH2, and male gonad tissue of oyster.

TABLE S1 The species that have been completed genome sequencing.

TABLE S2 Sequence information of collected SOHLH proteins.

TABLE S3 Titer detection in each period of preparation of SOHLH1 and SOHLH2 antibody (Unit: K).
