# Supplementary figures and images for: SOHLHs Might Be Gametogenesis-Specific bHLH Transcriptional Regulation Factors in Crassostrea gigas
Source: Front Physiol. 2019 May 15;10:594. doi: 10.3389/fphys.2019.00594 (PMC6529535; doi:10.3389/fphys.2019.00594)

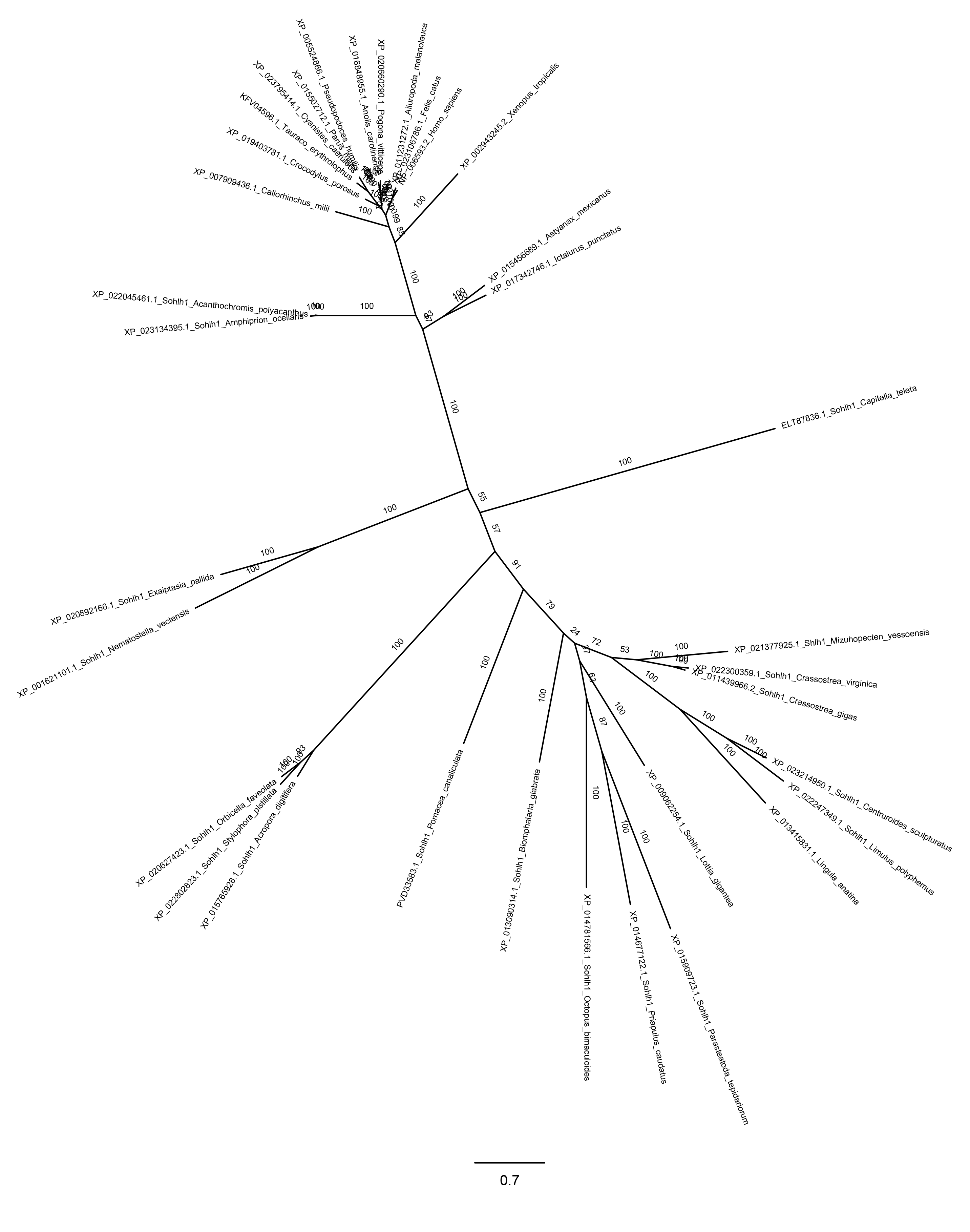

Supplement: FIGURE S1 — Phylogenetic analysis of SOHLH1 full sequence in species. [file Image_1.TIF]

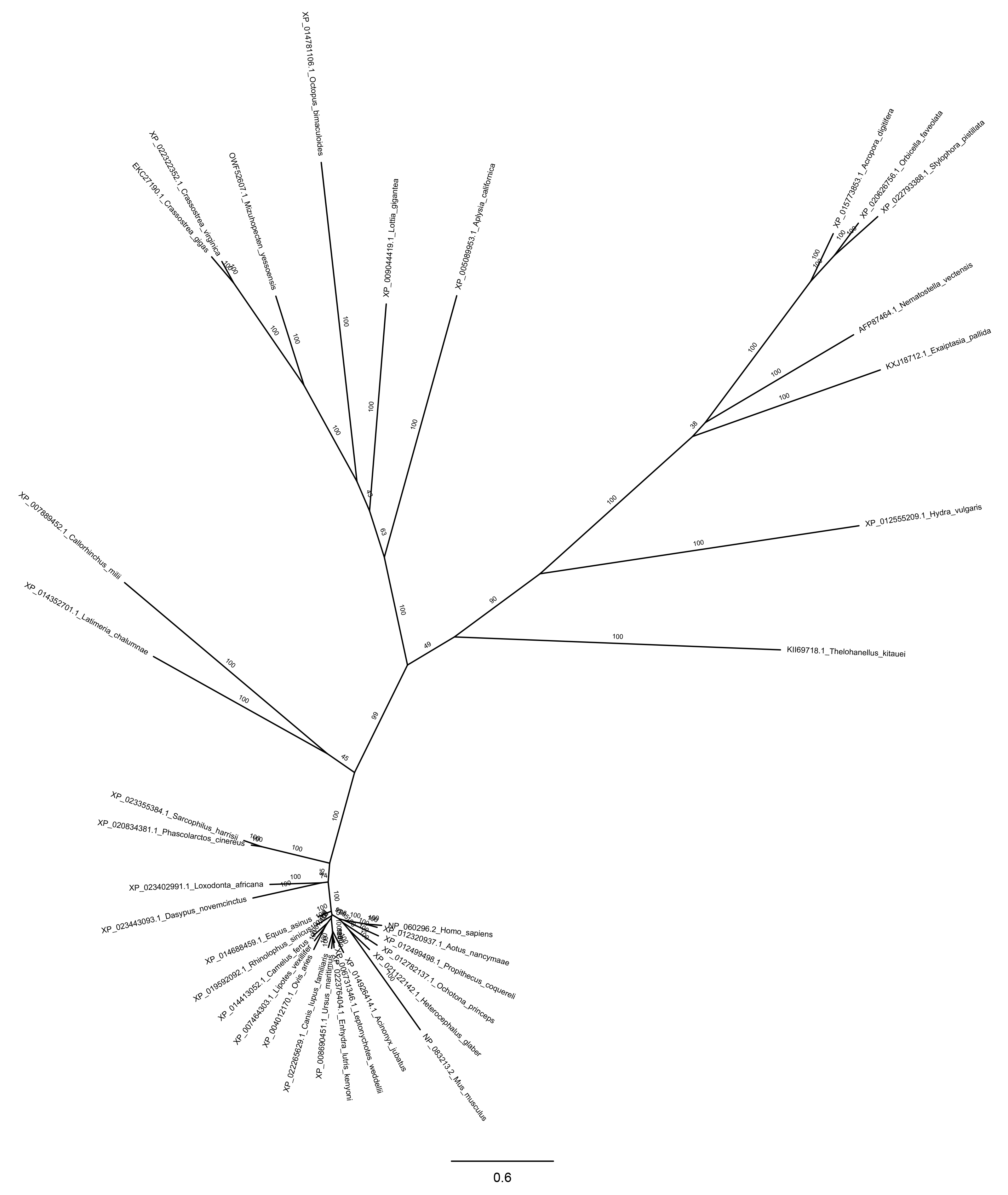

Supplement: FIGURE S2 — Phylogenetic analysis of SOHLH2 full sequence in species. [file Image_2.TIF]

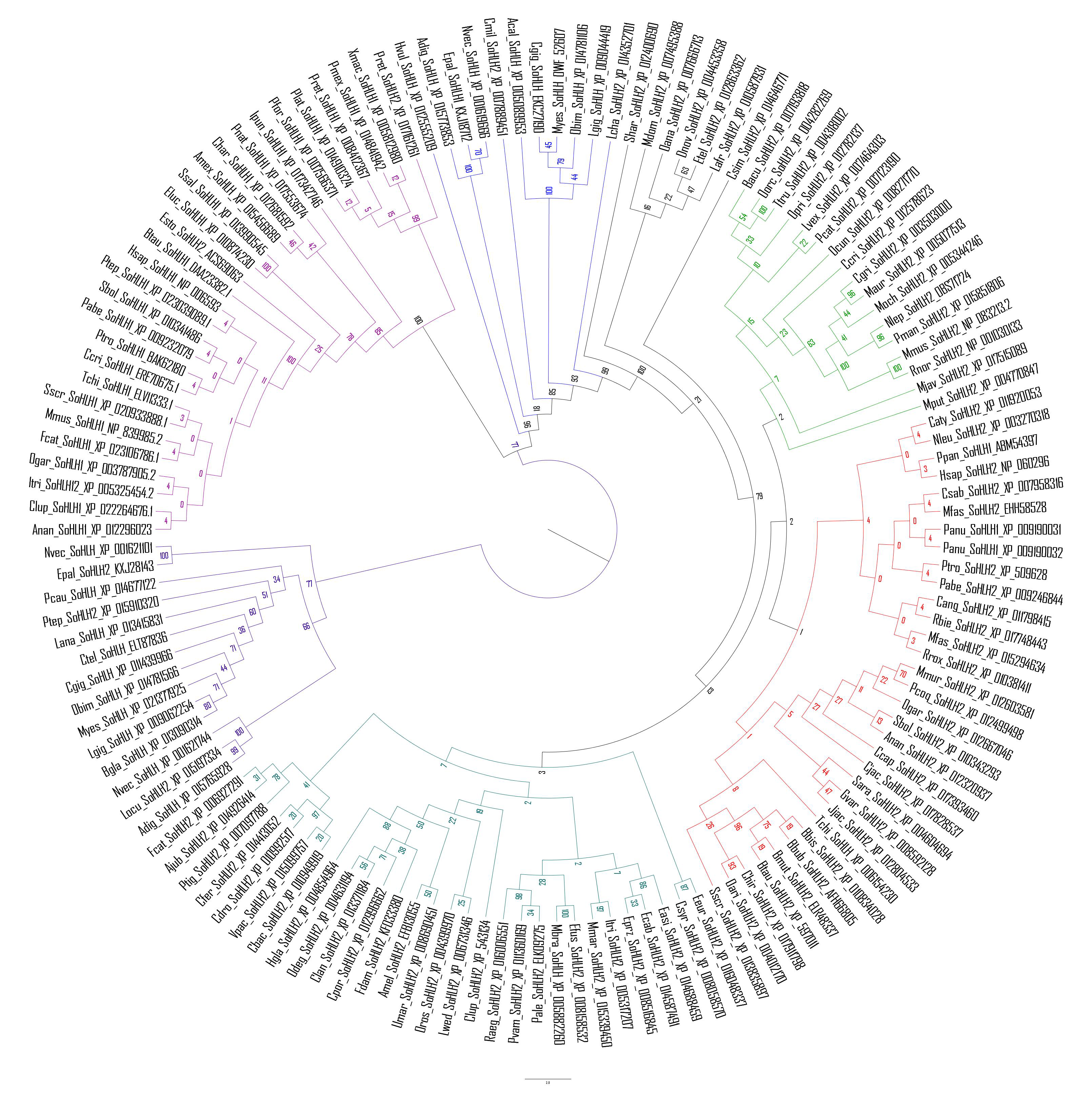

Supplement: FIGURE S3 — Phylogenetic analysis of bHLH domain of SOHLH1 and SOHLH2 in species. [file Image_3.TIF]

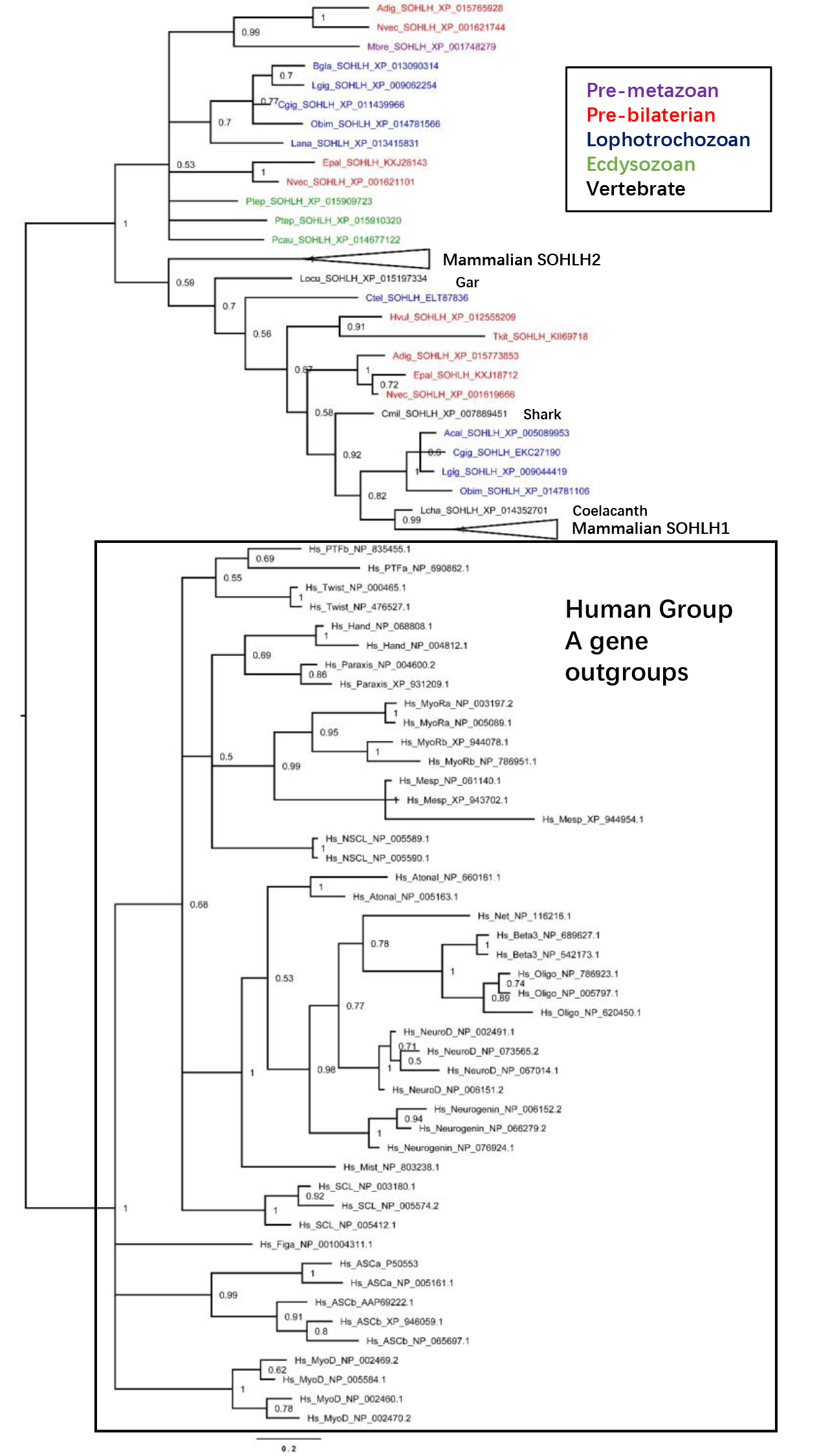

Supplement: FIGURE S4 — Phylogenetic analysis of bHLH domain of SOHLH1 and SOHLH2 in species with human group A gene outgroups according published article (Bao et al., 2017). [file Image_4.TIF]

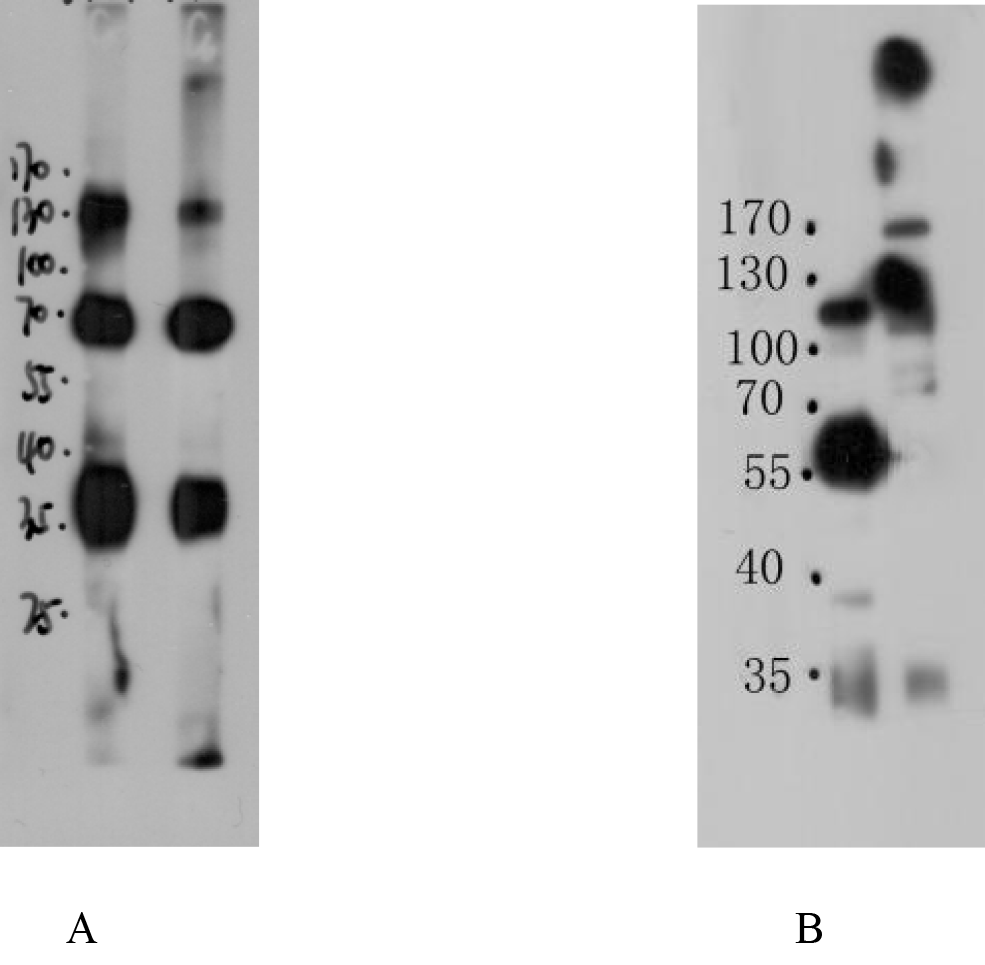

Supplement: FIGURE S5 — Western blot results of CgSOHLH1 and CgSOHLH1antibody detecting. (A) The target band of CgSOHLH1 is about 70 kDa. The bands from left to right are marker, recombinant protein and male gonad tissue of oyster. (B) The target band of Cg SOHLH2 is about 50 kDa. The bands from left to right are marker, recombinant protein of SOHLH2, and male gonad tissue of oyster. [file Image_5.TIF]
