# Supplementary material for: SOHLHs Might Be Gametogenesis-Specific bHLH Transcriptional Regulation Factors in Crassostrea gigas
Source: Front Physiol. 2019 May 15;10:594. doi: 10.3389/fphys.2019.00594 (PMC6529535; doi:10.3389/fphys.2019.00594)
Supplement: TABLE S1 — The species that have been completed genome sequencing. [file Table_1.DOCX]

**Table S1 the species that have been completed genome sequencing**

| Phylum | Class | Order | Family | Genus | Species |
| --- | --- | --- | --- | --- | --- |
| Protozoa | Aconoidasida | Haemosporida | Plasmodiidae | *Plasmodium* | *Plasmodium vivax* |
|  |  |  |  |  | *Plasmodium cynomolgi* |
|  |  |  |  |  | *Plasmodium reichenowi* |
|  |  |  |  |  | *Plasmodium malariae* |
|  |  |  |  |  | *Plasmodium reichenowi* |
|  | Sarcodina | Foraminiferida |  | *Reticulomyxa* | *Reticulomyxa filosa* |
| Euglenozoa | Kinetoplastida | Trypanosomatida |  | *Trypanosoma* | *Trypanosoma cruzi* |
| Placozoa |  |  | Trichoplacidae | *Trichoplax* | *Trichoplax adhaerens* |
| Porifera | Demospongiae | Haplosclerida | Niphatidae | *Amphimedon* | *Amphimedon queenslandica* |
| Cnidaria | Anthozoa | Actiniaria | Edwardsiidae | *Nematoctella* | *Nematostella vectensis* |
|  |  |  | Aiptasiidae | *Aiptasia* | *Aiptasia* |
|  |  | Scleractinia | Acroporidae | *Acropora* | *Acropora digitifera* |
|  | Hydrozoa | Anthoathecata | Hydridae | *Hydra* | *Hydra* |
|  | Myxosporea | Multivalvulida | Kudoidae | *Kudoa* | *Kudoa iwatai* |
| Ctenophora | Tentaculata | Lobata | Bolinopsidae | *Mnemiopsis* | *Mnemiopsis leidyi* |
| Platyhelminthes | Turbellaria | Macrostomida | Macrostomidae | *Macrostomum* | *Macrostomum lignano* |
|  | Cestoda | Cyclophyllidea | Taeniidae | *Echinococcus* | *Echinococcus multilocularis* |
|  |  |  |  |  | *Echinococcus granulosus* |
|  |  |  |  | *Taenia* | *Taenia asiatica* |
|  |  |  |  |  | *Taenia saginata* |
|  | Trematoda | Diplostomida | Schistosomatidae | *Schistosoma* | *Schistosoma mansoni* |
|  |  |  |  |  | *Schistosoma japonicum* |
|  |  |  |  |  | *Schistosoma haematobium* |
|  |  | Plagiorchiida | Opisthorchiidae | *Clonorchis* | *Clonorchis sinensis* |
|  |  |  |  | *Opisthorchis* | *Opisthorchis viverrini* |
| Rotifera | Bdelloidea | Bdelloida | Adinetidae | *Adineta* | *Adineta vaga* |
| Nematoda | Secernentea | Ascaridida | Toxocaridae | *Toxocara* | *Toxocara canis* |
|  |  | Strongylida | Trichostrongylidae | *Haemonchus* | *Haemonchus contortus* |
|  |  |  | Ancylostomatidae | *Necator* | *Necator americanus* |
|  |  | Spirurida | Onchocercidae | *Brugia* | *Brugia malayi* |
|  |  |  |  | *Onchocerca* | *Onchocerca volvulus* |
|  |  | Rhabditida | Rhabditidae | *Caenorhabditis* | *Caenorhabditis briggsae* |
|  |  |  |  |  | *Caenorhabditis elegans* |
|  |  | Tylenchida | Heteroderidae | *Meloidogyne* | *Meloidogyne incognita* |
|  | Chromadorea | Rhabditida | Ancylostomatidae | *Ancylostoma* | *Ancylostoma ceylanicum* |
|  |  | Ascaridida | Ascarididae | *Ascaris* | *Ascaris suum* |
|  | Enoplea | Trichurida | Trichuridae | *Trichuris* | *Trichuris* |
|  |  |  |  | *Trichinella* | *Trichinella spiralis* |
| Mollusca | Bivalvia | Mytiloida | Mytilidae | *Bathymodiolus* | *Bathymodiolus platifrons* |
|  |  |  |  | *Modiolus* | *Modiolus philippinarum* |
|  |  | Ostreoida | Pectinidae | *Patinopecten* | *Patinopecten yessoensis* |
|  |  |  | Ostreidae | *Magallana* | *Crassostrea gigas* |
|  | Gastropoda | Haliotoidea | Haliotidae | *Haliotis* | *Haliotis discus* |
|  |  | Lottioidea | Lottiidae | *Lottia* | *Lottia gigantea* |
|  | Cephalopoda | Octopoda | Octopodidae | *Octopus* | *Octopus bimaculoides* |
| Annelida | Clitellata | Rhynchobdellida | Glossiphoniidae | *Helobdella* | *Helobdella robusta* |
|  | Polychaeta |  | Capitellidae | *Capitella* | *Capitella teleta* |
| Arthropoda | Chilopoda | Geophilomorpha | Linotaeniidae | *Strigamia* | *Strigamia maritima* |
|  | Remipedia | Harpacticoida |  | *Tigriopus* | *Tigriopus kingsejongensis* |
|  | Branchiopoda | Cladocera | Daphniidae | *Daphnia* | *Daphnia pulex* |
|  | Arachnida | Trombidiformes | Tetranychidae | *Tetranychus* | *Tetranychus urticae* |
|  |  | Ixodida | Ixodidae | *Ixodes* | *Ixodes scapularis* |
|  |  | Sarcoptiformes | Sarcoptidae | *Sarcoptes* | *Sarcoptes scabiei* |
|  |  | Scorpiones | Buthidae | *Mesobuthus* | *Mesobuthus martensii* |
|  | Insecta | Orthoptera | Acrididae | *Locusta* | *Locusta migratoria* |
|  |  | Phthiraptera | Pediculidae | *Pediculus* | *Pediculus humanus humanus* |
|  |  | Isoptera | Termopsidae | *Zootermopsis* | *Zootermopsis nevadensis* |
|  |  | Homoptera | Aphididae | *Acyrthosiphon* | *Acyrthosiphon pisum* |
|  |  | Hemiptera | Delphacidae | *Nilaparvata* | *Nilaparvata lugens* |
|  |  |  | Cimicidae | *Cimex* | *Cimex lectularius* |
|  |  |  | Aleyrodidae | *Bemsia* | *Bemsia tabaci* |
|  |  | Coleoptera | Curculionidae | *Dendroctonus* | *Dendroctonus ponderosae* |
|  |  |  |  | *Hypothenemus* | *Hypothenemus hampei* |
|  |  |  | Tenebrionidae | *Tribolium* | *Tribolium castaneum* |
|  |  |  | Cerambycidae | *Anoplophora* | *Anoplophora glabripennis* |
|  |  | Hymenoptera | Apidae | *Apis* | *Apis mellifera* |
|  |  |  |  |  | *Apis florea* |
|  |  |  |  | *Bombus* | *Bombus terrestris* |
|  |  |  |  |  | *Bombus impatiens* |
|  |  |  |  | *Eufriesea* | *Eufriesea mexicana* |
|  |  |  |  | *Lasioglossum* | *Lasioglossum albipes* |
|  |  |  |  | *Megachile* | *Megachile rotundata* |
|  |  |  |  | *Melipona* | *Melipona quadrifasciata* |
|  |  |  |  | *Habropoda* | *Habropoda laboriosa* |
|  |  |  | Pteromalidae | *Nasonia* | *Nasonia vitripennis* |
|  |  |  |  |  | *Nasonia giraulti* |
|  |  |  |  |  | *Nasonia longicornis* |
|  |  |  | Formicidae | *Camponotus* | *Camponotus floridanus* |
|  |  |  |  | *Harpegnathos* | *Harpegnathos saltator* |
|  |  |  |  | *Linepithema* | *Linepithema humile* |
|  |  |  |  | *Pogonomyrmex* | *Pogonomyrmex barbatus* |
|  |  |  |  | *Solenopsis* | *Solenopsis invicta* |
|  |  |  |  | *Atta* | *Atta cephalotes* |
|  |  |  |  | *Acromyrmex* | *Acromyrmex echinatior* |
|  |  |  |  | *Cerapachys* | *Cerapachys biroi* |
|  |  |  |  | *Pseudomyrmex* | *Pseudomyrmex* |
|  |  | Lepidoptera | Bombycidae | *Bombyx* | *Bombyx mori* |
|  |  |  | Nymphalidae | *Danaus* | *Danaus plexippus* |
|  |  |  |  | *Melitaea* | *Melitaea cinxia* |
|  |  |  |  | *Heliconius* | *Heliconius melpomene* |
|  |  |  | Lycaenidae | *Calycopis* | *Calycopis* |
|  |  |  | Sphingidae | *Manduca* | *Manduca sexta* |
|  |  |  | Plutellidae | *Plutella* | *Plutella xylostella* |
|  |  |  | Papilionidae | *Papilio* | *Papilio glaucus* |
|  |  | Diptera | Calliphoridae | *Phormia* | *Phormia regina* |
|  |  |  | Tephritidae | *Ceratitis* | *Ceratitis capitata* |
|  |  |  |  | *Parochlus* | *Parochlus steinenii* |
|  |  |  | Chironomidae | *Antarctic* | *Antarctic midge* |
|  |  |  | Drosophilidae | *Drosophila* | *Drosophila serrata* |
|  |  |  |  |  | *Drosophila melanogaster* |
|  |  |  |  |  | *Drosophila pseudoobscura* |
|  |  |  |  |  | *Drosophila mauritiana* |
|  |  |  | Culicidae | *Anopheles* | *Anopheles gambiae* |
|  |  |  |  |  | *Anopheles gambiae* |
|  |  |  |  |  | *Anopheles darlingi* |
|  |  |  |  | *Aedes* | *Aedes aegypti* |
|  |  |  |  |  | *Aedes aegypti* |
|  |  |  |  |  | *Aedes albopictus* |
|  |  |  |  |  | *Aedes albopictus* |
|  |  |  |  | *Culex* | *Culex quinquefasciatus* |
| Tardigrada | Eutardigrada | Parachaela | Hypsibiidae | *Hypsibius* | *Hypsibius dujardini* |
|  |  |  |  | *Ramazzottius* | *Ramazzottius varieornatus* |
| Echinodermata | Holothuroidea | Synallactida | Stichopodidae | *Apostichopus* | *Apostichopus japonicus* |
|  | Echinoidea | Echinoida | Strongylocentrotidae | *Strongylocentrotus* | *Strongylocentrotus purpuratus* |
| Brachiopoda | Lingulata | Lingulida | Lingulidae | *Lingula* | *Lingula anatina* |
| Hemichordata | Enteropneusta | Enteropneusta | Harrimaniidae | *Saccoglossus* | *Saccoglossus*  *kowalevskii* |
|  |  |  | Ptychoderidae | *Ptychodera* | *Ptychodera flava* |
| Chordata | Hyperoartia | Petromyzontiformes | Petromyzontidae | *Petromyzon* | *Petromyzon marinus* |
|  | Ascidiacea | Enterogona | Cionidae | *Ciona* | *Ciona intestinalis* |
|  | Leptocardii | Copelata | Oikopleuridae | *Oikopleura* | *Oikopleura* |
|  |  | Amphioxiformes | Branchiostomidae | *Branchiostoma* | *Branchiostoma floridae* |
|  |  |  |  | *lancelet* | *lancelet* |
|  | Chondrichthyes | Chimaeriformes | Callorhinchidae | *Callorhinchus* | *Callorhinchus milii* |
|  | Actinopterygii | Beloniformes | Adrianichthyidae | *Oryzias* | *Oryzias latipes* |
|  |  |  |  | *Nothobranchiu* | *Nothobranchius furzeri* |
|  |  |  |  | *Protosalanx* | *Protosalanx hyalocranius* |
|  |  | Osmeriformes | Salangidae | *Protosalanx* | *Protosalanx hyalocranius* |
|  |  | Salmoniformes | Salmonidae | *Salmo* | *Salmo salar* |
|  |  | Gadiformes | Gadidae | *Gadus* | *Gadus morhua* |
|  |  | Osteoglossiformes | Osteoglossidae | *Scleropages* | *Scleropages formosus* |
|  |  | Siluriformes | Ictaluridae | *Ictalurus* | *Ictalurus punctatus* |
|  |  | Syngnathiformes | Syngnathidae | *Syngnathus* | *Syngnathus scovelli* |
|  |  |  |  | *Hippocampus* | *Hippocampus comes* |
|  |  | Gasterosteiformes | Gasterosteidae | *Gasterosteus* | *Gasterosteus aculeatus* |
|  |  | Cyprinodontiformes | Aplocheilidae | *Nothobranchius* | *Nothobranchius furzeri* |
|  |  |  | Poeciliidae | *Xiphophorus* | *Xiphophorus maculatus* |
|  |  |  | Cyprinidae | *Cyprinus* | *Cyprinus carpio* |
|  |  |  |  | *grass* | *grass carp* |
|  |  |  |  | *Danio* | *Danio rerio* |
|  |  | Anabantiformes | Channidae | *Channa* | *Channa argus* |
|  |  |  |  | *Thunnus* | *Thunnus orientalis* |
|  |  | Perciformes | Nototheniidae | *Notothenia* | *Notothenia coriiceps* |
|  |  |  | Stromateidae | *Pampus* | *Pampus argenteus* |
|  |  |  | Carangidae | *Seriola* | *Seriola quinqueradiata* |
|  |  |  | Sciaenidae | *Larimichthys* | *Larimichthys crocea* |
|  |  |  | Sciaenidae | *Larimichthys* | *Larimichthys crocea* |
|  |  | Tetraodontiformes | Molidae | *Mola* | *Mola mola* |
|  |  |  | Tetraodontidae | *Fugu* | *Fugu rubripes* |
|  |  |  |  | *Tetraodon* | *Tetraodon nigroviridis* |
|  |  |  |  | *Tetraodontidae* | *Tetraodontidae* |
|  |  | Sarcopterygii | Latimeriidae | *Latimeria* | *Latimeria chalumnae* |
|  |  | Pleuronectiformes | Paralichthyidae | *Paralichthys* | *Paralichthys olivaceus* |
|  |  |  | Cynoglossidae | *Cynoglossus* | *Cynoglossus semilaevis* |
|  |  | Lepisosteiformes | Lepisosteidae | *Lepisosteus* | *Lepisosteus oculatus* |
|  |  | Cypriniformes | Cyprinidae | *Sinocyclocheilus* | *Sinocyclocheilus* |
|  | Amphibia | Urodela | Cryptobranchidae | *Cryptobranchus* | *Cryptobranchus alleganiensis* |
|  |  | Anura | Dicroglossidae | *Nanorana* | *Nanorana parkeri* |
|  |  |  |  | *Xenopus* | *Xenopus tropicalis* |
|  |  | Testudines | Emydidae | *Chrysemys picta* | *Chrysemys picta bellii* |
|  |  |  | Trionychidae | *Pelodiscus* | *Pelodiscus sinensis* |
|  |  |  | Cheloniidae | *Chelonia* | *Chelonia mydas* |
|  |  | Crocodilia | Crocodylidae | *Crocodylus* | *Crocodylus siamensis* |
|  |  |  | Alligatoridae | *Alligator* | *Alligator mississippiensis* |
|  |  |  |  |  | *Alligator sinensis* |
|  | Reptilia | Squamata | Viperidae | *Deinagkistrodon* | *Deinagkistrodon acutus* |
|  |  |  | Eublepharidae | *Eublepharis* | *Eublepharis macularius* |
|  |  |  | Elapidae | *Ophiophagus* | *Ophiophagus hannah* |
|  |  |  | Colubridae | *Pantherophis* | *Pantherophis guttatus* |
|  |  |  | Pythonidae | *Python* | *Python molurus bivittatus* |
|  |  |  |  | *Gekko* | *Gekko japonicus* |
|  |  |  | Dactyloidae | *Anolis* | *Anolis carolinensis* |
|  |  |  | Agamidae | *Pogona* | *Pogona vitticeps* |
|  | Aves | Galliformes | Phasianidae | *Gallus* | *Gallus sonneratii* |
|  |  |  |  | *Meleagris* | *Meleagris gallopavo* |
|  |  | Passeriformes | Estrildidae | *Taeniopygia* | *Taeniopygia guttata* |
|  |  |  | Thraupidae | *Geospiza* | *Geospiza fortis* |
|  |  |  | Muscicapidae | *Ficedula* | *Ficedula albicollis* |
|  |  |  | Paridae | *Pseudopodoces* | *Pseudopodoces humilis* |
|  |  |  |  |  | *Parus humilis* |
|  |  |  |  |  | *Parus major* |
|  |  |  | Fringillidae | *Serinus* | *Serinus canaria* |
|  |  |  | Acanthisittidae | *Acanthisitta* | *Acanthisitta chloris* |
|  |  |  | Corvidae | *Corvus* | *Corvus brachyrhynchos* |
|  |  |  | Pipridae | *Manacus* | *Manacus vitellinus* |
|  |  | Otidiformes | Otidae | *Chlamydotis* | *Chlamydotis macqueenii* |
|  |  | Eurypygiformes | Eurypygidae | *Eurypyga* | *Eurypyga helias* |
|  |  | Psittaciformes | Psittaculidae | *Melopsittacus* | *Melopsittacus undulatus* |
|  |  |  |  | *Ara* | *Ara macao* |
|  |  |  | Nestoridae | *Nestor* | *Nestor notabilis* |
|  |  | Columbiformes | Columbidae | *Columba* | *Columba livia* |
|  |  | Falconiformes | Falconidae | *Falco* | *Falco peregrinus* |
|  |  |  |  |  | *Falco cherrug* |
|  |  |  |  |  | *Falco peregrinus* |
|  |  | Accipitriformes | Accipitridae | *Haliaeetus* | *Haliaeetus albicilla* |
|  |  |  |  |  | *Haliaeetus leucocephalus* |
|  |  |  | Cathartidae | *Cathartes* | *Cathartes aura* |
|  |  | Anseriformes | Anatidae | *Anas* | *Anas platyrhynchos* |
|  |  |  | Anatidae | *goose* | *goose* |
|  |  | Ciconiiformes | Threskiorothidae | *Egretta* | *Egretta garzetta* |
|  |  | Musophagiformes | Musophagidae | *Tauraco* | *Tauraco erythrolophus* |
|  |  | Phoenicopteriformes | Phoenicopteridae | *Phoenicopterus* | *Phoenicopterus ruber* |
|  |  | Trogoniformes | Trogonidae | *Apaloderma* | *Apaloderma vittatum* |
|  |  | Sphenisciformes | Spheniscidae | *Aptenodytes* | *Aptenodytes forsteri* |
|  |  |  |  | *Pygoscelis* | *Pygoscelis adeliae* |
|  |  | Gruiformes | Gruidae | *Balearica* | *Balearica regulorum* |
|  |  | Cariamiformes | Cariamidae | *Cariama* | *Cariama cristata* |
|  |  | Mesitornithiformes | Mesitornithidae | *Mesitornis* | *Mesitornis unicolor* |
|  |  | Bucerotiformes | Bucerotidae | *Buceros* | *Buceros rhinoceros* |
|  |  | Leptosomiformes | Leptosomidae | *Leptosomus* | *Leptosomus discolor* |
|  |  | Coraciiformes | Meropidae | *Merops* | *Merops nubicus* |
|  |  | Apodiformes | Trochilidae | *Calypte* | *Calypte anna* |
|  |  |  | Apodidae | *Chaetura* | *Chaetura pelagica* |
|  |  | Charadriiformes | Scolopacidae | *Philomachus* | *Philomachus pugnax* |
|  |  |  | Charadriidae | *Charadrius* | *Charadrius vociferus* |
|  |  | Coliiformes | Coliidae | *Colius* | *Colius striatus* |
|  |  | Cuculiformes | Cuculidae | *Cuculus* | *Cuculus canorus* |
|  |  | Opisthocomiformes | Opisthocomidae | *Ophisthocomus* | *Ophisthocomus hoazin* |
|  |  | Procellariiformes | Procellariidae | *Fulmarus* | *Fulmarus glacialis* |
|  |  | Gaviiformes | Gaviidae | *Gavia* | *Gavia stellata* |
|  |  | Pelecaniformes | Pelecanidae | *Pelecanus* | *Pelecanus crispus* |
|  |  | Suliformes | Phalacrocoracidae | *Phalacrocorax* | *Phalacrocorax carbo* |
|  |  | Phaethontiformes | Phaethontidae | *Phaethon* | *Phaethon lepturus* |
|  |  | Podicipediformes | Podicipedidae | *Podiceps* | *Podiceps cristatus* |
|  |  | Struthioniformes | Struthionidae | *Struthio* | *Struthio camelus* |
|  |  | Pteroclidiformes | Pteroclididae | *Pterocles* | *Pterocles gutturalis* |
|  |  | Piciformes | Picidae | *Picoides* | *Picoides pubescens* |
|  |  | Strigiformes | Tytonidae | *Tyto* | *Tyto alba* |
|  |  | Tinamiformes | Tinamidae | *Tinamus* | *Tinamus guttatus* |
|  |  | Caprimulgiformes | Caprimulgidae | *Antrostomus* | *Antrostomus carolinensis* |
|  |  | Apterygiformes | Apterygidae | *Apteryx* | *Apteryx* |
|  | Mammalia | Monotremata | Ornithorhynchidae | *Ornithorhynchus* | *Ornithorhynchus anatinus* |
|  |  | Diprotodontia | Macropodidae | *Macropus* | *Macropus eugenii* |
|  |  | Dasyuromorphia | Dasyuridae | *Sarcophilus* | *Sarcophilus harrisii* |
|  |  | Artiodactyla | Lipotidae | *Lipotes* | *Lipotes vexillifer* |
|  |  |  | Balaenidae | *Balaena* | *Balaena mysticetus* |
|  |  | Proboscidea | Elephantidae | *Mammuthus* | *Mammuthus primigenius* |
|  |  | Perissodactyla | Equidae | *Equus* | *Equus caballus* |
|  |  |  |  |  | *Equus asinus* |
|  |  | Artiodactyla | Bovidae | *Bison* | *Bison bonasus* |
|  |  |  |  |  | *Bos taurus* |
|  |  |  |  |  | *Bos grunniens* |
|  |  | Artiodactyla | Bovidae | *Capra* | *Capra hircus* |
|  |  |  |  | *Ovis* | *Ovis aries* |
|  |  |  |  | *Pantholops* | *Pantholops hodgsonii* |
|  |  |  | Giraffidae | *Giraffa* | *Giraffa camelopardalis* |
|  |  |  |  | *Okapia* | *Okapia johnstoni* |
|  |  |  | Camelidae | *Camelus* | *Camelus bactrianus* |
|  |  |  | Suidae | *Sus* | *Sus scrofa* |
|  |  | Lagomorpha | Lepus | *rabbit* | *rabbit* |
|  |  | Carnivor | Ailuridae | *Ailurus* | *Ailurus fulgens* |
|  |  |  | Canidae | *Canis* | *Canis familiaris* |
|  |  |  |  | *Vulpes* | *Vulpes vulpes* |
|  |  |  | Felidae | *Felis* | *Felis catus* |
|  |  |  |  | *Panthera* | *Panthera pardus orientalis* |
|  |  |  |  | *Panthera* | *Panthera tigris* |
|  |  |  |  | *Acinonyx* | *Acinonyx jubatus* |
|  |  |  | Ursidae | *Ailuropoda* | *Ailuropoda melanoleura* |
|  |  |  |  | *Ursus* | *Ursus maritimus* |
|  |  |  | Mustelidae | *Mustela* | *Mustela putorius furo* |
|  |  | Rodentia | Castoridae | *Castor* | *Castor canadensis* |
|  |  |  | Muridae | *Rattus* | *Rattus norvegicus* |
|  |  |  |  | *Mus* | *Mus musculus* |
|  |  |  | Cricetidae | *Cricetulus* | *Cricetulus griseus* |
|  |  |  | Spalacidae | *Spalax* | *Spalax galili* |
|  |  |  |  | *Heterocephalus* | *Heterocephalus glaber* |
|  |  | Pholidota | Manidae | *Manis* | *Manis pentadactyla* |
|  |  |  |  |  | *Manis javanica* |
|  |  | Chiroptera | Pteropodidae | *Pteropus* | *Pteropus alecto* |
|  |  |  | Vespertilionidae | *Myotis* | *Myotis davidii* |
|  |  |  |  |  | *Myotis brandtii* |
|  |  | Didelphimorphia | Didelphidae | *Monodelphis* | *Monodelphis domestica* |
|  |  | Primates | Hominidae | *Gorilla* | *Gorilla* |
|  |  |  |  | *Pongo* | *Pongo abelii* |
|  |  |  |  |  | *Pongo pygmaeus* |
|  |  |  |  | *Gorilla* | *Gorilla gorilla gorilla* |
|  |  |  |  | *Pan* | *Pan troglodytes* |
|  |  |  |  |  | *Pan paniscus* |
|  |  |  | Cercopithecidae | *Rhinopithecus* | *Rhinopithecus* |
|  |  |  |  | *Rhinopithecus* | *Rhinopithecus bieti* |
|  |  |  |  | *Macaca* | *Macaca mulatta* |
|  |  |  |  |  | *Macaca fascicularis* |
|  |  |  |  |  | *Macaca mulatta lasiota* |
|  |  |  | Lemuridae | *Eulemur* | *Eulemurﬂavifrons* |
|  |  |  | Daubentoniidae | *Daubentonia* | *Daubentonia madagascariensis* |
|  |  |  | Callitrichidae | *Marmoset* | *Marmoset* |
|  |  |  | Hylobatidae | *Nomascus* | *Nomascus leucogenys* |
|  |  |  | Tarsiidae | *Tarsius* | *Tarsius syrichta* |
|  |  | Scandentia | Tupaiidae | *Tupaia* | *Tupaia belangeri* |
|  |  | Dermoptera | Cynocephalidae | *Galeopterus* | *Galeopterus variegatus* |
