# Supplementary material for: SOHLHs Might Be Gametogenesis-Specific bHLH Transcriptional Regulation Factors in Crassostrea gigas
Source: Front Physiol. 2019 May 15;10:594. doi: 10.3389/fphys.2019.00594 (PMC6529535; doi:10.3389/fphys.2019.00594)
Supplement: TABLE S2 — Sequence information of collected SOHLH proteins. [file Table_2.DOCX]

**Table S2 Sequence information of collected Sohlh proteins**

| Gene | Species | ID | Species | ID |
| --- | --- | --- | --- | --- |
| Sohlh1 | *Amazona aestiva* | KQL12465.1 | *Anolis carolinensis* | XP_016848955.1 |
|  | *Aotus nancymaae* | XP_012296023 | *Astyanax mexicanus* | XP_015456689 |
|  | *Balaenoptera acutorostrata scammoni* | XP_007198713.1 | *Canis lupus familiaris* | XP_022264676.1 |
|  | *Cariama cristata* | KFP67114.1 | *Cervus elaphus hippelaphus* | OWK03028.1 |
|  | *Chlamydotis macqueenii* | KFP45603.1 | *Chrysemys picta bellii* | XP_005302950.2 |
|  | *Clupea harengus* | XP_012681592 | *Colius striatus* | KFP32056.1 |
|  | *Corvus brachyrhynchos* | KFO53283.1 | *Crocodylus porosus* | XP_019403781.1 |
|  | *Cyanistes caeruleus* | XP_023795414.1 | *Enhydra lutris kenyoni* | XP_022349664.1 |
|  | *Eptatretus stoutii* | ACS69063 | *Esox lucius* | XP_010874230 |
|  | *Felis catus* | XP_023106786.1 | *Homo sapiens* | NP_006593 |
|  | *Ictalurus punctatus* | XP_017342746 | *Manacus vitellinus* | KFW75508.1 |
|  | *Meleagris gallopavo* | XP_010720796.1 | *Miniopterus natalensis* | XP_016057987.1 |
|  | *Nestor notabilis* | KFQ41765.1 | *Odobenus rosmarus divergens* | XP_004410192.1 |
|  | *Oncorhynchus mykiss* | XP_021422330.1 | *Ophiophagus hannah* | ETE61944.1 |
|  | *Pan troglodytes* | BAK62180 | *Panthera tigris altaica* | XP_007087082.1 |
|  | *Parus major* | XP_015502712.1 | *Pelodiscus sinensis* | XP_006126268.1 |
|  | *Picoides pubescens* | KFV62525.1 | *Poecilia formosa* | XP_007566371 |
|  | *Poecilia latipinna* | XP_014910324 | *Poecilia reticulata* | XP_017161261 |
|  | *Pogona vitticeps* | XP_020660290.1 | *Pongo abelii* | XP_009232079 |
|  | *Pseudopodoces humilis* | XP_005524866.1 | *Pygocentrus nattereri* | XP_017553674 |
|  | *Salmo salar* | XP_013990545 | *Salvelinus alpinus* | XP_023861789.1 |
|  | *Saimiri boliviensis* | XP_010341486 | *Sinocyclocheilus grahami* | XP_016110902.1 |
|  | *Sorex araneus* | XP_012790868.1 | *Tauraco erythrolophus* | KFV04596.1 |
|  | *Tupaia chinensis* | ELV11333.1 | *Xenopus tropicalis* | XP_016110902.1 |
|  | *Xiphophorus maculatus* | XP_005812980 |  |  |
| Sohlh2 | *Acinonyx jubatus* | XP_014926414 | *Acropora digitifera* | XP_015773853 |
|  | *Aotus nancymaae* | XP_012320937 | *Balaenoptera acutorostrata scammoni* | XP_007193818 |
|  | *Bos taurus* | XP_597011 | *Callithrix jacchus* | XP_017828537 |
|  | *Callorhinchus milii* | XP_007889451 | *Camelus ferus* | XP_014413052 |
|  | *Canis lupus familiaris* | XP_543134 | *Capra hircus* | XP_017911798 |
|  | *Cebus capucinus imitator* | XP_017393460 | *Chinchilla lanigera* | XP_013371184 |
|  | *Crassostrea gigas* | EKC27190 | *Eptesicus fuscus* | XP_008158532 |
|  | *Equus asinus* | XP_014688459 | *Equus przewalskii* | XP_008516845 |
|  | *Erinaceus europaeus* | XP_016048337 | *Exaiptasia pallida* | KXJ18712 |
|  | *Heterocephalus glaber* | XP_004854964 | *Homo sapiens* | NP_060296 |
|  | *Latimeria chalumnae* | XP_014352701 | *Lepisosteus oculatus* | XP_015197334 |
|  | *Leptonychotes weddellii* | XP_006731346 | *Lipotes vexillifer* | XP_007464303 |
|  | *Macaca fascicularis* | XP_005585693 | *Mustela putorius furo* | XP_004770847 |
|  | *Nomascus leucogenys* | XP_003270318 | *Ochotona princeps* | XP_012782137 |
|  | *Odobenus rosmarus diverge* | XP_004399970 | *Orcinus orca* | XP_004282269 |
|  | *Ornithorhynchus anatinus* | XP_007666713 | *Pan troglodytes* | XP_509628 |
|  | *Panthera tigris altaica* | XP_007097788 | *Physeter catodon* | XP_007123190 |
|  | *Priapulus caudatus* | XP_014677122 | *Saimiri boliviensis boliviensis* | XP_010343293 |
|  | *Sarcophilus harrisii* | XP_012400690 | *Sus scrofa* | XP_013835897 |
|  | *Ursus maritimus* | XP_008690451 |  |  |
| uncharacterized | *Acropora digitifera* | XP_015765928 | *Aplysia californica* | XP_005089953 |
|  | *Biomphalaria glabrata* | XP_013090314 | *Capitella teleta* | ELT87836 |
|  | *Centruroides sculpturatus* | XP_023214950.1 | *Crassostrea gigas* | XP_011439966 |
|  | *Exaiptasia pallida* | KXJ28143 | *Hydra vulgaris* | XP_012555209 |
|  | *Limulus polyphemus* | XP_022247349.1 | *Lingula anatina* | XP_013415831 |
|  | *Lottia gigantea* | XP_009044419 | *Lottia gigantea* | XP_009062254 |
|  | *Nematostella vectensis* | XP_001621101 | *Nematostella vectensis* | XP_001621744 |
|  | *Octopus bimaculoides* | XP_014781106 | *Octopus bimaculoides* | XP_014781566 |
|  | *Parasteatoda tepidariorum* | XP_015909723 | *Parasteatoda tepidariorum* | XP_015910320 |
|  | *Stylophora pistillata* | XP_022802823.1 |  |  |
