# Supplementary material for: SOHLHs Might Be Gametogenesis-Specific bHLH Transcriptional Regulation Factors in Crassostrea gigas
Source: Front Physiol. 2019 May 15;10:594. doi: 10.3389/fphys.2019.00594 (PMC6529535; doi:10.3389/fphys.2019.00594)
Supplement: TABLE S3 — Titer detection in each period of preparation of SOHLH1 and SOHLH2 antibody (Unit: K). [file Table_3.docx]

Table S3 Titer detection in each period of preparation of SOHLH1 and SOHLH2 antibody (Unit: K).

| Antibody | each period of preparation | A rabbit | B rabbit | C rabbit | D rabbit |
| --- | --- | --- | --- | --- | --- |
| SOHLH1 | Third immunization | 512 | 256 | 512 | 256 |
|  | Fourth immunization | 512 | 512 | 512 | 512 |
|  | Final serum | 512 | 512 | 512 | 512 |
|  | Purified antibody | 512 | 512 | 512 | 512 |
| SOHLH2 | Third immunization | 512 | 128 | 512 | 512 |
|  | Fourth immunization | 512 | 512 | 512 | 512 |
|  | Final serum | 512 | 512 | 256 | 512 |
|  | Purified antibody | 128 | 128 | 32 | 128 |

Standard of antibody titer: ≥512.
